# Estimating snow water equivalent from GPS vertical site-position observations in the western United States

**DOI:** 10.1002/wrcr.20173

**Published:** 2013-05-28

**Authors:** Karli J Ouellette, Caroline de Linage, James S Famiglietti

**Affiliations:** 1 Department of Earth System Science, University of CaliforniaIrvine, Irvine, California, USA; 2 UC Center for Hydrologic Modeling, University of CaliforniaIrvine, Irvine, California, USA

## Abstract

[1] Accurate estimation of the characteristics of the winter snowpack is crucial for prediction of available water supply, flooding, and climate feedbacks. Remote sensing of snow has been most successful for quantifying the spatial extent of the snowpack, although satellite estimation of snow water equivalent (SWE), fractional snow covered area, and snow depth is improving. Here we show that GPS observations of vertical land surface loading reveal seasonal responses of the land surface to the total weight of snow, providing information about the stored SWE. We demonstrate that the seasonal signal in Scripps Orbit and Permanent Array Center (SOPAC) GPS vertical land surface position time series at six locations in the western United States is driven by elastic loading of the crust by the snowpack. GPS observations of land surface deformation are then used to predict the water load as a function of time at each location of interest and compared for validation to nearby Snowpack Telemetry observations of SWE. Estimates of soil moisture are included in the analysis and result in considerable improvement in the prediction of SWE.

**Citation:** Ouellette, K. J., C. de Linage, and J. S. Famiglietti (2013), Estimating snow water equivalent from GPS vertical site-position observations in the western United States, *Water Resour. Res.*, *49*, 2508–2518, doi:10.1002/wrcr.20173.

## 1 Introduction and Background

[2] Snowpack characteristics of primary importance to hydrologists and water managers include snow depth, density, snow water equivalent (SWE), and areal extent. Of these, the combination of SWE with areal extent provides critical information about the volume of water stored as snow in a particular region. Snow depth and density may be best measured using traditional, ground-based methods at present, and areal extent can be well monitored from space because of the high reflectivity of snow [*Dozier*, 1989; *Kelly et al*., 2003; *Painter et al*., 2003]. Remote sensing of SWE remains an important challenge, owing to the need to characterize both snow depth and density, or the mass of water stored within the snowpack [*Alsdorf et al*., 2007]. The Gravity Recovery and Climate Experiment (GRACE) [*Tapley et al*., 2004] mission has proven skillful at monitoring water mass changes [e.g., *Swenson and Wahr*, 2006; *Rodell et al*., 2009; *Famiglietti et al*., 2011], including those dominated by snow [e.g., *Frappart et al*., 2006], ice sheets, and glaciers [e.g., *Tamisiea et al*., 2007; *Velicogna and Wahr*, 2006a,2006b; *Velicogna*, 2009; *Jacob et al*., 2012], yet the low spatiotemporal resolution of GRACE (monthly, >200,000 km^2^) limits its utility for smaller-scale, subregional applications as well as for submonthly mass changes.

[3] Beyond GRACE, other space geodetic measurements, such as altimetry, have proven fruitful when applied to hydrology. For example, altimetric measurements of snow depth [*Papa et al*., 2002; *Deems et al*., 2006; *Hopkinson et al*., 2004; *Hall and Riggs*, 2007], of inland surface water bodies [*Calmant et al*., 2008] and of river discharge [*Smith*, 1997] all show great promise [*Durand et al*., 2010]. Observations of vertical land surface displacement from interferometric synthetic aperture radar have been related to poroelastic responses to water storage changes within aquifer systems [*Galloway et al*., 1998; *Amelung et al*., 1999]. While GPS has enabled an ability to constrain the elastic parameters of the solid Earth [*Bevis et al*., 2005; *Steckler et al*., 2010], the potential for GPS to invert the hydrological load and constrain some important hydrological parameters remains largely untapped, even though many studies show a clear correlation at various spatial scales between GPS vertical and horizontal position solutions and water storage changes or their predicted deformation [*Davis et al*., 2004; *Tregoning et al*., 2009; *Ji and Herring*, 2012]. Very few studies have been devoted to snow-load-induced deformation at GPS stations [*Heki*, 2001; *Grapenthin et al*., 2006]. Recently, *Larson et al*. [2008,^2009^], *Gutmann et al*. [2011], and *Larson et al*. [2012] demonstrated the capabilities for monitoring soil moisture and snowpack using GPS multipaths, while *Bevis et al*. [1992] and several others have shown its utility for monitoring atmospheric water vapor. *Meertens et al*. [2008] began to explore the hydrologic contributions to land surface displacement observed by GPS.

[4] In this study, we explore the utility of GPS for quantifying SWE. We first demonstrate that observed vertical displacements at several GPS stations in the western United States are dominated by elastic loading induced by the snowpack and soil moisture. We then use independent data to determine appropriate parameters for the analytical, 2-D response of the elastic half-space model of *Tsai* [2011]. We use this model to predict water storage variations at six locations in the western United States with good results. Our findings suggest that the portion of the global GPS network located in snow-prone climates has good potential for measuring SWE.

[5] Permanent GPS stations were originally installed throughout the western United States to observe tectonic plate motion and seismic activity, as well as to support surveyors and local government agencies. As significant seasonal variations in the site-position time series became apparent [*vanDam and Herring*, 1994; *vanDam et al*., 1994; *Blewitt et al*., 2001; *MacMillan and Ma*, 2000; *vanDam et al*., 2001; *Mangialotti et al*., 2001], efforts were made to model the various nonseismic contributions to the seasonal variations and remove them from the GPS time series. Potential contributions from pole tide effects, ocean tide loading, atmospheric loading, nontidal ocean mass, and groundwater loading were evaluated and modeled by *Dong et al*. [2002] for several GPS stations globally. The results indicate that significant seasonal variations are expected, particularly in response to pole tide variations. The importance of each contribution depends on the station location, with ocean loading increasing toward the coasts and atmospheric loading being larger at high latitudes.

[6] In addition to the seasonal elastic loading contributions addressed by *Dong et al*. [2002], *Prawirodirdjo et al*. [2006] investigated potential seasonal thermoelastic strain contributions to GPS time series in the Los Angeles Basin. Thermoelastic strain was modeled using the thermoelastic strain model of *Ben-Zion and Leary* [1986]. Results showed that the phase of the seasonal variations in GPS observations was well represented by the thermoelastic model. Expected site-position displacements were calculated by integrating thermoelastic strain in a follow-up study by *Tsai* [2011]. While the phase of the observed seasonal variations correlated with modeled displacements, the amplitude of modeled displacement was able to account for no more than 25% of the observed amplitude in the Los Angeles (LA) Basin.

[7] *Tsai* [2011] also analyzed the potential contribution of water storage changes to land surface position. Compaction and expansion of aquifers resulting from poroelastic strain caused by groundwater fluctuations has been observed with GPS site-position time series [*Bawden et al*., 2001; *Ji and Herring*, 2011]. In addition to poroelastic strain, the weight of groundwater and surface water also exert pressure on the land surface resulting in direct elastic strain. Following the methods from *Ben-Zion and Leary* [1986] and *Berger* [1975], poroelastic strain and direct elastic loading were modeled for the Los Angeles Basin. *Tsai* [2011] found that these hydrologic effects were able to explain the full amplitude of the seasonal changes observed by GPS in one location and concluded that poroelastic and elastic loading from water storage changes are the dominant contributors to the seasonality of the GPS signal in the basin.

[8] This study investigates the contribution of the seasonal snowpack to the observed land surface deformation through direct elastic loading. This work builds on that of *Tsai* [2011] by applying it to the inversion of SWE variations from GPS observed land surface deformation at six stations across the western United States. The expected contributions of thermoelastic and hydrologic loading to land surface displacement are calculated using the equations for an elastic half-space presented by *Tsai* [2011]. By characterizing the hydrologic load contribution, the potential for using GPS observations of seasonal land surface deformation for snowpack monitoring will be evaluated. Predictions of SWE are inverted from GPS observations and compared to Snowpack Telemetry (SNOTEL) observations [*Crook*, 1977]. The predictions are then improved by accounting for soil moisture variations observed by SNOTEL.

## 2. GPS and SNOTEL Observations

**Figure 1:**
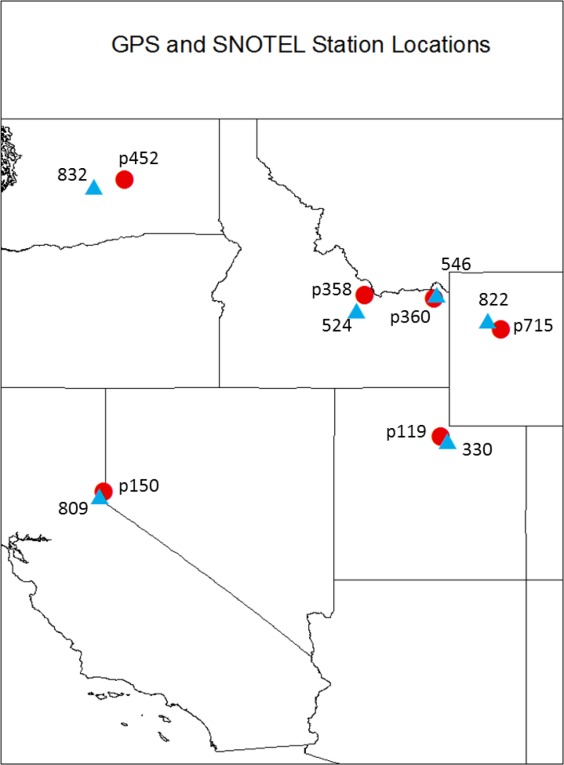
Six GPS stations from the SOPAC network and the nearest SNOTEL observation stations were selected in the western United States. The locations of each GPS station used in this study are marked in red. SNOTEL stations are marked in blue. [Color figure can be viewed in the online issue, which is available at wileyonlinelibrary.com.]

**Figure 2:**
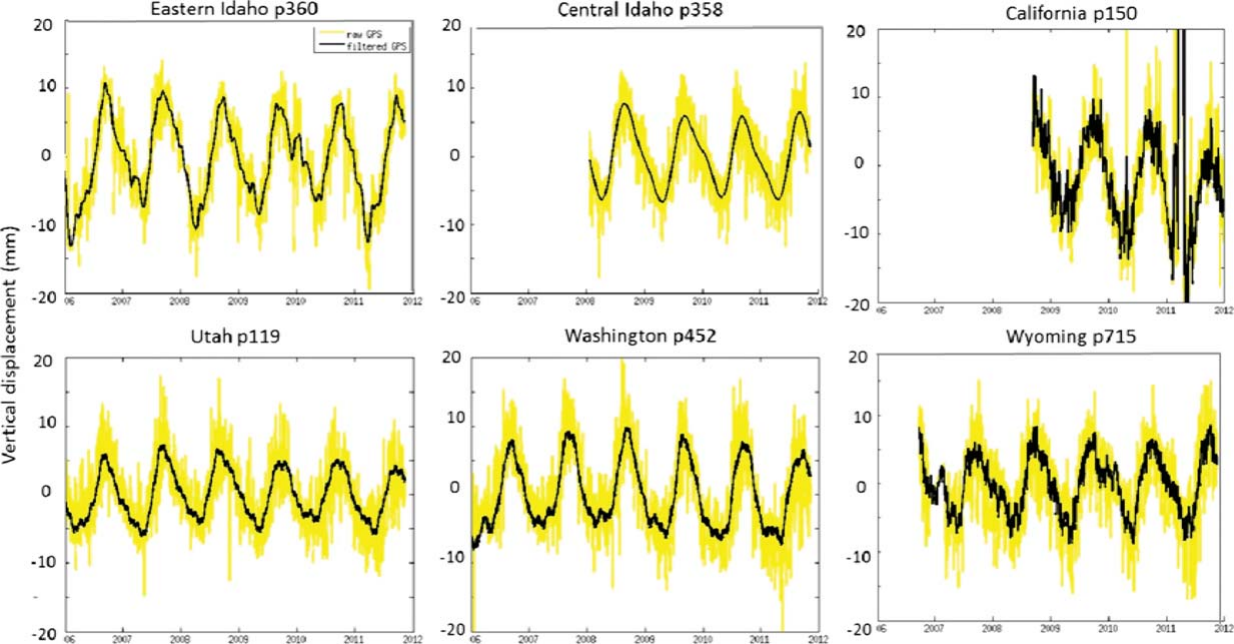
Raw GPS time series from SOPAC are shown in yellow. Low-pass filtered, detrended GPS data are shown in black.

[9] GPS observations were obtained from the network of 1100 GPS stations by Scripps Orbit and Permanent Array Center (SOPAC). GPS vertical time series and uncertainty estimates from the Plate Boundary Observatory (PBO) network, the geodetic component of EarthScope [*Rundle et al*., 2002], were processed using a SOPAC refined model with input derived from GAMIT and GLOBK software calculations [*Dongchen et al*., 2005; *Nikolaidis*, 2002]. Oscillations from well understood sources such as pole tide variation have been removed by the SOPAC refined model. The annual harmonic oscillation was estimated simultaneously with the trend using the least squares method. GPS vertical land surface position time series were chosen from six stations in the western United States. Stations were selected for proximity to available snow pack and soil moisture data with available time series of at least 2 years. Areas of significant documented aquifer poroelastic responses to groundwater depletion were avoided. Selected stations include p360 in eastern Idaho, p358 in central Idaho, p150 in eastern California, p452 in central Washington, p119 in northern Utah, and p715 in western Wyoming and are shown in Figure 1 and Table 1. Raw and low-pass filtered (discussed in section 3.3) time series of the GPS vertical position time series are shown in Figure 2. At every station we observe a strong annual variation, with the maximum subsidence (uplift) occurring in late winter (summer), respectively.

**Table 1:** Location of GPS Stations and Distance and Elevation Change Between Each Station and the Selected SNOTEL Site

	Location	Elevation (m)	SNOTEL Site	SNOTEL Location	Estimated Distance to SNOTEL Site (km)	Elevation Change to SNOTEL Site (m)
Idaho p360	44.32°N, 111.45°W	1858	Crab Creek 424	44.43°N, 112°W	9 (±5)	71
Idaho p358	44.4°N, −113.24°W	2420	Hilts Creek524	44.02°N, −113.47°W	55 (±5)	6
California p150	39.29°N, 120.03°W	2619	Tahoe City Cross 809	39.17°N, 120.15°W	22 (±5)	−167
Utah p119	40.73°N, −111.26°W	2046	Beaver Divide 330	40.62°N, −111.1°W	22 (±5)	146
Washington p452	47.4°N, −119.49°W	323	Trough 832	47.23°N, 120.3°W	93 (±5)	411
Wyoming p715	43.5°N, 109.69°W	2988	Togwotee Pass 822	43.75 N, −110.05°W	46 (±5)	−21

**Figure 3:**
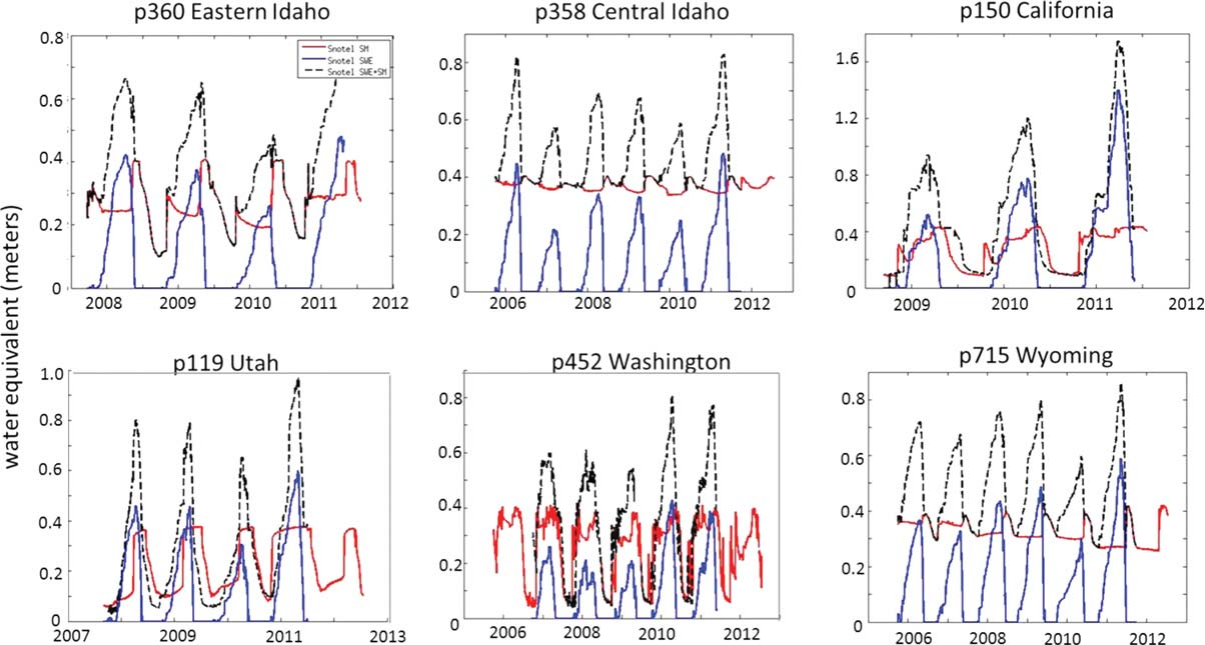
SNOTEL observations of soil moisture (red) and SWE (blue), and their sum (dashed black) for each location shown in Figure 1.

[10] SWE records were obtained from nearby SNOTEL sites [*Crook*, 1977, available at http://www.wcc.nrcs.usda.gov/snow]. SNOTEL records are obtained from in situ observations of a variety of climatic and hydrologic variables by the extensive, automated system run by the Natural Resources Conservation Service. In situ observations of SWE were converted to load pressure by multiplying the annual amplitude extracted from the observations by gravity and the density of water at standard temperature and pressure. Records of soil moisture were also obtained from SNOTEL. Soil water data are available as pore water content detected via automated Hydraprobe sensors installed at 2″, 8″, and 20″ depth. Total soil moisture water content was calculated as a sum of three layers of soil, 2″, 6″, and 126″ in thickness, each represented by a single Hydraprobe sensor. The observed pore water content at each measurement depth was used to calculate the soil moisture water equivalent for each soil layer using an assumed effective porosity of 0.3. The weighted average of these layers was then applied to an assumed total soil profile 3.43 ± 1 m in depth, following the Community Land Model [*Oleson et al*., 2010]. Daily SNOTEL records of SWE and soil moisture are shown in Figure 3. The annual amplitude of soil moisture variations as a percentage of the annual amplitude of SWE is highly variable between stations, ranging from 14% in California to 70% in Washington.

## 3. Methods

[11] The contribution of the snow load at each station in Figure 1 to land surface displacement was determined using the equations described by *Tsai* [2011]. Each equation expresses the 2-D response of an elastic half-space to a sinusoidal (both in time and in space) surface forcing. By representing the thermal and hydrologic signal as an annual sinusoidal function based on the observed annual harmonic, the annual harmonic response of the land surface can be predicted. Finally, the relationship between land surface deformation and SWE can be determined and applied to GPS observations to predict SWE for each location.

### 3.1. Model Equations

[12] The contributions of thermoelastic strain and seasonal water storage changes to land surface position were modeled following the solutions found by *Tsai* [2011] for an elastic half-space. Vertical thermoelastic strain 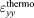 is expressed by *Tsai* [2011] as
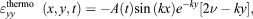
(1)with
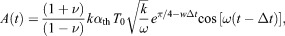
(2)where *k* is the horizontal wave number representing the spatial distribution of the thermal load (1-D along direction *x*), *ν* is Poisson’s ratio, *α*_th_ is the coefficient of linear thermal expansion, *T*_0_ is half of the peak-to-peak amplitude in temperature variation at frequency *ω*, *κ* is the thermal diffusivity, and Δ*t* is the time delay between the strain and the load. Integrating the thermoelastic strain over the depth *y* of the half-space gives the expression for surface vertical displacement:
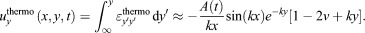
(3)

[13] Similarly, the expression for elastic strain from *Tsai* [2011, equation (9)] was used and integrated to model the effects of hydrologic loading on vertical land surface position such that
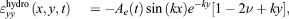
(4)
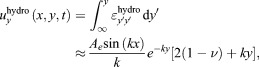
(5)
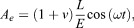
(6)where 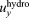 is the vertical displacement taken positive downward, *E* is Young’s modulus, and *L* is the amplitude of the total water storage load pressure at frequency *ω*.

[14] Equations (3) and (5) are applied to the observed annual temperature and total water storage observations, respectively, to calculate the expected annual land surface response to each forcing. The calculated land surface response is then compared to observed GPS records to determine the relative annual contribution of each forcing to the final observed displacement.

### 3.2. Choice of Parameters

**Figure 4:**
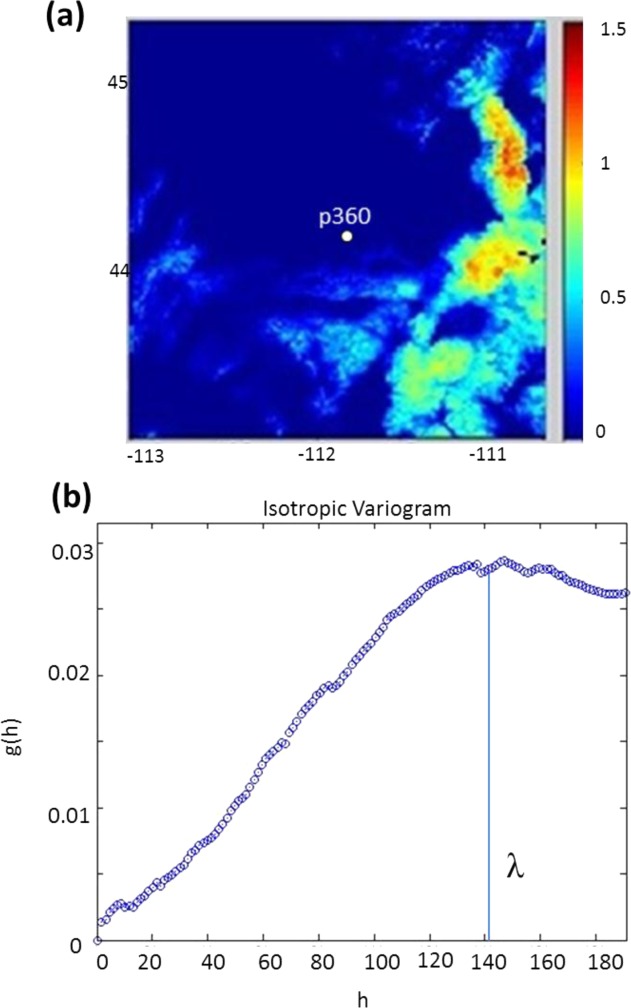
(a) Idaho SNODAS SWE data shown in meters and (b) spatial variogram calculated for SNODAS SWE data. The wavelength λ is inferred from the distance at which the variogram levels off.

[15] To apply the 2-D elastic equations at a variety of locations, parameters which are appropriate to each station must be determined. Where possible, observed and modeled data were used to determine local parameters specific to each location. An annual frequency (2 × 10^−7^ s^−1^) was chosen for this study. Values for thermal diffusivity (10^−6^ m^2^/s), and linear thermal expansion (10^−5^ °C^−1^) are not expected to vary considerably and are taken from *Tsai* [2011] for consistency. The horizontal wave number *k*, representing the spatial distribution of the load, was calculated for each location from 2 × 2 degree maps of 1 km resolution SWE blended model-observation product, the Snow Data Assimilation System (SNODAS) model [*National Operational Hydrologic Remote Sensing Center*, 2004]. Spatial variograms of the SNODAS data were calculated for each of the six locations to determine the characteristic wave length *λ* in the north–south, east–west, northwest–southeast, and southwest–northeast directions of the snow load (Figure 4) during the winter snow months as described by *Clark* [1979]. The wave number is then determined as 2*π*/*λ*. An average wave number was determined in each direction from several winter variograms, and the direction with the minimum wavelength was selected. The error is estimated as the standard deviation of winter variograms in the selected direction (Table 2). When variograms were not readable in all directions, the average of all readable variograms was used. The corresponding wave number was used for both thermoelastic and elastic strain calculations, following *Tsai* [2011]. The estimated spatial wavelengths shown in Table 2 are similar in scale to the spatial resolution of CRUST2.0 so that the spatial dimension of both the load and the model parameters are consistent.

**Table 2:** Estimated Crustal and Load Parameters From CRUST2.0 and SNODAS for Each GPS Station

	Young’s Modulus and Corresponding Error (GPa)	Poisson’s Ratio and Corresponding Error	Spatial Wavelength and Corresponding Error (km)
Idaho p360	100.3 (±13.6)	0.262 (±0.03)	104 (±8)
Idaho p358	98.3 (±13.6)	0.262 (±0.03)	95 (±8)
California p150	83.8 (±19.3)	0.257 (±0.03)	110 (±7)
Utah p119	86.6 (±13.6)	0.263 (±0.03)	60 (±9)
Washington p452	85.3 (±17.8)	0.252 (±0.03)	51 (±13)
Wyoming p715	77.4 (±14.4)	0.266 (±0.03)	57 (±7)

[16] Values for Poisson’s ratio and Young’s modulus were estimated from an exponentially weighted average of CRUST2.0 model parameters [*Bassin et al*., 2000, available at http://igppweb.ucsd.edu/∼gabi/crust2.html]—decreasing in weight with depth up to a maximum depth equal to the load characteristic wavelength for their respective locations. Crustal properties for the 2 × 2 degree grid which corresponds to each GPS station location were extracted from the model to estimate a unique parameter for each location using the equations described later.

[17] Using observed P-wave velocities, S-wave velocities, and crustal density for each layer of the crust estimated for each location from CRUST2.0, Poisson’s ratio was calculated by
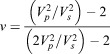
(7)and Young’s modulus from
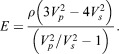
(8)

[18] Values for *E* and *ν* at each station are given in Table 2.

[19] The errors for *E* and *ν* were determined from either the difference between the values calculated for the six locations and the model average value from CRUST2.0 for continental crust, or the difference between the values calculated using an arithmetical average of the full crust and an exponentially decreasing average of crustal layers. The larger of these two errors was selected for each location. The resulting uncertainties are thus conservative estimates and are large enough to account for errors due to the propagation of uncertainties on *k* onto the averages and *E* and *ν*.

### 3.3. Water Storage Prediction

[20] After examining the relative contribution of each forcing to the annual land surface displacement, it is possible to determine a linear relationship based on the above equations between hydrologic load and land surface deformation which can be used for predictive purposes. In order to use GPS observations as a predictor of SWE, it is necessary to invert the relationship between the observed SWE and predicted land surface response, and apply this to the observed land surface response to calculate SWE. As shown later, the relationship determined for the annual frequency may be applied to the full range of frequencies in the GPS data which are caused by water storage variation. Very high frequency variations in the GPS signal may be attributed to noise and are not of interest to this study. In order to remove high frequency noise in the GPS signal unrelated to hydrologic effects, the GPS data were low-pass filtered to remove all periods smaller than 23 days, thus removing the intramonthly variability in the data. The long-term trend in GPS may also be attributed to isostatic or tectonic processes and is also removed. The raw GPS time series and the low-pass filtered, detrended GPS data are shown in Figure 2. Thermoelastic strain was assumed to be negligible as shown in section 4. According to Tsai’s model, the linear relationship between observed water storage and the land surface displacement is expressed as a rearrangement of equations (5) and (6):
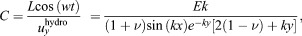
(9)where *L* cos(*ωt*) is the total water storage at a given frequency (e.g., annual), *u_y_*_,hydro_ is the induced land surface displacement at the same frequency. The expression for *C* can be further simplified by our assumption that *y* = 0, and *x* = *λ*/4 to represent the maximum of the sinusoid where *x* is centered at the station and *λ* is the wavelength. Thus,
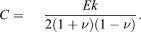
(10)

[21] Assuming that the wave number *k* is not dependent on frequency, *C* is an admittance, i.e., is frequency independent and may be applied to the sum of all frequencies of the load. The parameter *C* is therefore multiplied to the low-pass filtered observed land surface displacement from GPS to arrive at predicted water storage anomalies (WSA)
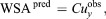
(11)where WSA^pred^ is the predicted SWE and 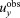 is the vertical land surface deformation observed by GPS.

## 4. Results

### 4.1. Annual Component of Vertical Surface Displacement

**Figure 5:**
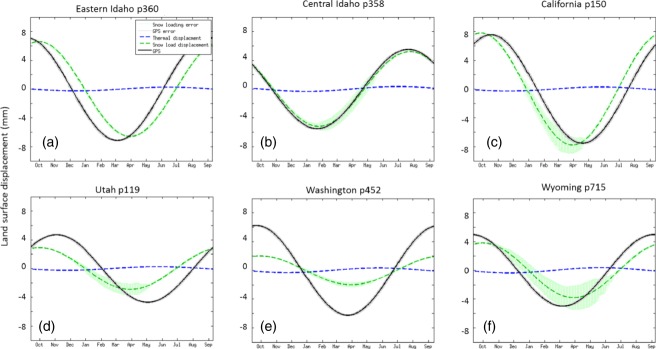
Annual harmonic in land surface displacement from modeled thermoelastic strain (blue), modeled snow loading (green), modeled snow loading error (light green), GPS observations (black), and GPS observation error (gray) in (a) Eastern Idaho, (b) Central Idaho, (c) California, (d) Utah, (e) Washington, and (f) Wyoming.

[22] The annual amplitude of vertical surface deformation estimated at the six stations (Figure 5) ranges from 4.7 (station p119) to 7.5 mm (station p150). The results of this study (shown in Table 2 and Figure 5) reveal that the snow-load-induced deformation is enough to explain the phase as well as 32%–103% of the amplitude of the GPS observed annual land surface vertical displacement in all six locations. The deformation induced by the thermoelastic effect has an annual amplitude much smaller (4%–7%) than that of GPS observed annual deformation and lags the peak of observed deformation by 3 months. Therefore, because thermoelastic strain is not the primary cause for the observed annual deformation observed by GPS, it is excluded in our remaining analyses.

[23] The predicted annual variations in land surface height from the snow load account for 92% of the observed land surface variation at the eastern Idaho station, 94% at the central Idaho station, 103% at the California station, and 76% of the variation at the station in Wyoming. The amplitude of land surface deformation is significantly underpredicted in Utah, 62%, and Washington, 32%. Differences in phase are also shown in Table 3. Large differences in phase may be attributable to elevation changes between the GPS and SNOTEL stations, as well as contributions of soil moisture to the surface load which are discussed in section 4.3. The discrepancies between modeled and observed vertical displacements may also be due to errors in load parameterization as well as parameter estimation for *ν* or *E* for those locations. Note that our focus was on obtaining representative parameters from independent data sources, and not site-specific parameter estimation, which surely would improve the correspondence shown in Figure 5.

**Table 3:** Values Are Given for the Percent of Observed Annual Land Surface Deformation That Is Predicted by Thermoelastic and Elastic Strain, as Well as the Difference in Phase Between the Observed and Predicted Annual Land Surface Deformation

GPS Station	Thermal Contribution (%)	SWE Contribution (%)	SWE Contribution Phase Difference (days)
Idaho p360	4	92	−35
Idaho p358	6	94	−21
California p150	4	103	24
Washington p452	5	32	−17
Utah p119	6	62	28
Wyoming p715	7	76	−29

[24] A perfect fit is not expected to occur due to the generally small but significant distance and elevation change between the GPS and SNOTEL stations shown in Table 1, particularly at the Washington location where stations in close proximity were not available. A difference in spatial-scale sensitivity also affects each observation type. SNOTEL stations represent a singular point observation which responds to highly localized water storage, while GPS observations integrate the effect of a wider spatially distributed water storage load on the land surface.

**Figure 6:**
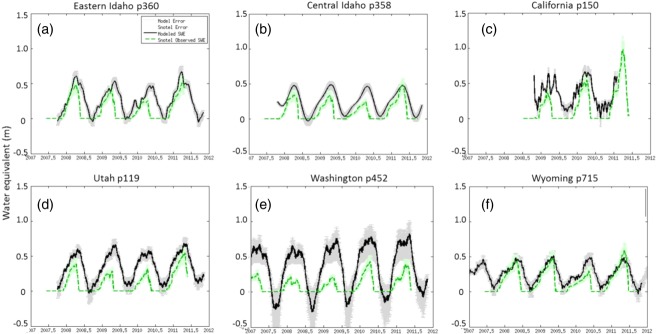
Snow water equivalent variations predicted by GPS observations (black) and model error (gray) and observed by SNOTEL (green) and observation error (light green) in (a) Eastern Idaho, (b) Central Idaho, (c) California, (d) Utah, (e) Washington, and (f) Wyoming.

**Figure 7:**
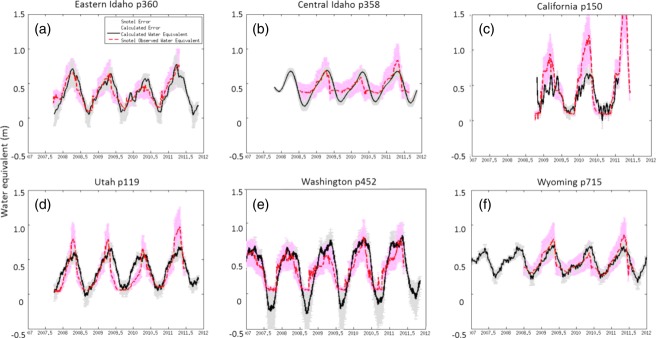
Total water storage variations predicted by GPS observations (black) and model error (gray) and observed by SNOTEL (red) and observation error (pink) in (a) Eastern Idaho, (b) Central Idaho, (c) California, (d) Utah, (e) Washington, and (f) Wyoming.

[25] A range of values was considered for each model parameter to produce the errors shown in Figures 6 and 7. The range considered for *E* and *ν*, as described in section 3.3, is at least as large as the maximum difference between the values calculated for each location and the model average for continental crust. This should account for possible errors resulting from the spatial variability of crustal parameters not accounted for due to the spatial scale of the load being similar in size to the grid size of CRUST2.0. An error of 20% was also assumed for the SNODAS SWE, to account for both measurement error and spatial variability in the snowpack, though the spatial variability is unknown. Likewise, the error for total water equivalent was assumed to be 30% to account for the additional uncertainty in the contribution of soil water content. It should be noted that the results may represent an overestimation as we assumed the maximum load at *λ*/4 for our calculations. Overall, the results here suggest that SWE loading is a dominant driver of seasonal land surface deformation in these regions.

[26] The crustal response to water storage variations as well as the water load at the six locations may have nonnegligible energy at frequencies other than the annual frequency. The case for this is further developed when a wider range of frequencies is used for comparison in the next section of this paper.

### 4.2. Using GPS Vertical Displacements to Predict Snow Load Variations

[27] As described in section 3.3, according to Tsai’s model, we determined the admittance between calculated vertical land surface deformation and the hydrologic load following equation (10). This relationship is then used to predict SWE variations at each site using GPS observations of land surface deformation.

[28] Since this relationship is not frequency dependent, it may be applied to all frequencies contained in the observations, though periodic variations less than 23 days in the GPS observations are likely to be dominated by nonhydrologic phenomena. For this reason, this method is useful for monitoring the accumulation of snow throughout the winter season but is not useful for observing short-term responses to individual precipitation events. The predicted SWE variation is shown and compared to observed water storage variation in Figure 6, with normalized root-mean-square deviation (NRMSD) between the predicted and observed water storage for each location displayed in Table 4. Errors in the modeled SWE were propagated from the estimated error in each model parameter.

**Table 4:** NRMSD Between Observed and Predicted SWE, and SWE + Soil Moisture

GPS Station	NRMSD of Predicted SWE (%)	NRMSD of Predicted Total Water Storage
Idaho p360	42	13
Idaho p358	65	32
California p150	61	21
Washington p452	147	19
Utah p119	72	15
Wyoming p715	34	15

[29] Variations in SWE at a range of frequencies appear to correlate with variations in modeled SWE at all six locations. SWE is reasonably well predicted by GPS in eastern Idaho (NRMSD of 42%), as well as the Wyoming location (NRMSD of 34%). Problems with the GPS time series in California correspond to the unusually deep snowpack in 2011, causing a significant perturbation of the GPS signal presumably by burial when the SWE exceeds 1 m depth at this site, as can be seen in Figures 2 and 3. Similar effects of snow covering the pillar and/or the dome protecting the antenna have been observed on the GPS signal by *Jaldehag et al*. [1996] and modeled by *Webb et al*. [1995]. Because those effects bias the height measurements, data from late winter 2011 were excluded from the analysis at the California station. Taller GPS stations may be necessary for SWE observation in deep snowpack regions. The SWE is significantly overpredicted by GPS in Utah and Washington (NRMSD of 72% and 147%, respectively), which suggested that the spatial scale of the snow load to which GPS measurements are sensitive is not local but regional, and that other loading sources, such as soil moisture, are contributing to the observed land surface motion. Likewise, all six stations poorly represent the spring snowmelt.

[30] In California and Wyoming, there appear to be semiannual variations in the GPS record which are unrelated to the hydrologic load, perhaps as a result of processing errors [*Tregoning and Watson*, 2009; *Ray et al*., 2008]. As a result, prediction of semiannual water storage variations in these locations is less accurate. Accurate modeling and removal of the unknown contributions could potentially improve prediction of water storage at high frequencies.

### 4.3. Contribution of Soil Moisture to Water Load Predicted From GPS

[31] To account for the discrepancies between the observed and predicted snowmelt, the contribution of soil moisture water storage was added to the analysis. SNOTEL measurements of pore water content from the nearest available stations were converted to soil water equivalent, using assumptions about the soil type and depth, described in section 2. SNOTEL SWE and soil water equivalent were summed to produce a time series of total water storage equivalent (Figure 3). Since the soil depth is poorly characterized, an error of ±1 m of soil depth was assumed. The total water storage equivalent was then compared to the GPS predicted water storage load in Figure 7. The NRMSD values between observed and modeled total water storage are given in Table 4.

[32] When soil moisture is included in the analysis, significant improvement in the prediction of the spring melt is observed at every station, as shown by the decrease of the NRMSD values at those stations (Table 4). This suggests that melting snow in spring is absorbed by the thawing soil before draining to lakes and rivers. A recovery of the land surface after winter loading by the snowpack does not occur until the subsequent draining of water from the soil. This suggests that GPS data are useful in observing not only the buildup of the winter snowpack, but also the gradual release of snow water in the spring. In Washington and Utah, significantly larger variations in soil moisture are observed than at the other locations used in this study, leading to the strongest NRMSD reductions. SWE may be accurately predicted in isolation only in locations where soil deposits are thin or well drained, as appears to be the case in Idaho and Wyoming.

[33] Deviation in the prediction of water storage variation in Washington compared to the observed water storage may also be a result of the greater spatial separation (93 km) and elevation difference (1347 m) of the GPS and SNOTEL stations in this location which can be seen in Figure 1 and Table 1. The anomalous underprediction of water storage by GPS compared to SNOTEL observations in 2010 may be the result of a snowdrift or local snow accumulation which affected the SNOTEL station, but does not translate to the more spatially integrated GPS prediction. The results in Idaho, Utah, and Wyoming suggest that the full range of frequencies in recorded water storage variation results in a response in the vertical land surface measurable by GPS (NRMSD of 13%–32%). Overall, these results suggest that GPS has the capability to predict water storage variations at a spatial scale at least on the order of the distance between GPS and SNOTEL stations at these locations (up to 46 km).

## 5. Discussion

[34] Networks of continuous GPS receiver stations are expanding worldwide. The information regarding water storage contained in the GPS vertical time series is valuable for water storage assessment, including SWE in inaccessible regions. There are several advantages inherent to a GPS observation technique. GPS may be installed and automated for remote access, providing high temporal resolution data while eliminating the need for extensive field expeditions. Permanent GPS stations are likely to remain more robust than automated snow pillow detection methods and less vulnerable to errors caused by snow drifts. Due to the integrative response of the Earth’s crust, the load inferred from observations of surface vertical deformation is integrated over a larger area less vulnerable to errors caused by snow drifts. Since the land surface at a given point is responding to a surface load over a larger area related to the wave number described earlier, the predicted water storage from GPS observations will take into account the spatial distribution of the load, minimizing the effects of highly localized anomalies. Our method provides regional estimates of uniform snow cover over scales of 60–120 km.

[35] The regional scale of the snow cover estimates given by the present method is also an advantage over the use of multipaths providing a local (approximately 1000 m^2^ around a GPS receiver) estimate of the snow cover [*Larson et al*., 2009; *Larson and Nievinski*, 2012]. On the other hand, GPS offers a smaller-scale observation of water mass changes than the GRACE mission, allowing for applications on regional scales.

[36] Discrepancies in the predicted and observed water storage at all locations may also be a result of differences in the local snowpack at the SNOTEL and GPS stations. Though the nearest stations were chosen for comparison, the distance between stations may allow for significant variations in the water storage because snow pack can exhibit high spatial variability. A shift in amplitude and phase may be expected due to a change in elevation between stations depending on the location of the thawing line. Both issues of elevation change and spatial heterogeneity will affect the comparison in Washington (refer to Table 1). Additionally, SNOTEL observations from snow pillows respond to a 1 m^2^ area and may be affected by snow drifts or other local anomalies, while GPS observations of the crustal deformation respond to a spatially integrated load over a larger area [*Molotch et al*., 2005; *Molotch and Bales*, 2006; *Meromy et al*., 2012]. Other contributing factors may be a poor estimate of soil porosity and other estimated parameters, a simplified 1-D load geometry, as well as model errors.

[37] Estimates of the horizontal wave number *k* in this study are based on the correlation length of SNODAS models of SWE in a sampling of directions. Improved estimates of *k* could be made by more thorough accounting for the 2-D distribution of the snow load as well as assuming a different value of *k* for the soil moisture component using high quality observations of soil moisture, since both soil moisture and snow act as surface loads on different spatial scales. For the purposes of this study, a spatial correlation between soil moisture and SWE is assumed. A dynamic estimate of *k* which varies each year may improve the representation of interannual variability in the snowpack.

[38] The comparison of water storage predicted by GPS data to measured SNOTEL data is also limited by large uncertainty in the measured soil moisture. SNOTEL measurements of pore water content are limited to the first 20″ of soil. Extrapolation of these measurements for deeper soil is not possible given the very small number of measurement points (three). The depth of the soil profile and the porosity of the soil are also unknown, leading to a large uncertainty in total soil moisture as can be seen in the uncertainty in Figure 7. Error bars were estimated based on 3.43 ± 1 m of soil depth. Even with uncertainty, the prediction is significantly improved when soil moisture is included in the analysis at every station (Table 4). However, even with the uncertainty, the simulations fail to reproduce the observations in spring 2009 for both the California and Utah locations, meaning that an important contribution is missing in the model. The proximity of Lake Tahoe to GPS station p150 in California is a likely contributor to this error.

[39] Overall, the good agreement found between calculated and observed water equivalent indicates that the values chosen for the model parameters are reasonable. The results show that the land surface responds to water storage loading on seasonal, annual, and interannual timescales, and the simple technique developed here can be used to accurately estimate variations in water storage on these timescales using GPS time series and modeled parameters.

[40] Further work is needed to determine an appropriate density of GPS observations needed to accurately quantify the water storage variation in a region of interest. The burial of GPS receivers under snow, as seen in the GPS observations from p150 in California, requires strategic planning to minimize occurrences. Horizontal land surface motion recorded by GPS may also be used to infer information about the 2-D load distribution. Soil moisture detection by GPS multipath techniques developed by *Larson et al*. [2010] may be incorporated with this technique as a method to separate soil moisture variations from SWE. Finally, water storage estimations from GPS could be integrated into existing data assimilation models such as the SNODAS to improve current estimates of winter snowpack.

## 6. Summary

[41] The results of this study indicate that the snowpack and soil moisture load dominate the seasonal GPS vertical displacement signal at mountain sites in the western United States. Using the 2-D equations built by *Tsai* [2011] for a half-space model subjected to a 1-D load, the CRUST2.0 model parameters, as well as load spatial extent from SNODAS data, it was determined that hydrologic loading dominates the seasonal land surface deformation observed by GPS, while thermoelastic strain has a negligible effect. The relationship between land surface deformation and SWE is then used to predict SWE from GPS time series with reasonable accuracy (13%–32% NRMSD). GPS thus has potential as a hydrologic observation tool for monitoring of SWE at regional, spatial scales in environments with minimal soil moisture variation. In regions with significant soil water storage variation, GPS can be used to observe the sum of SWE and soil moisture, with separation of variables possible with use of auxiliary techniques. This technique serves to advance current capabilities for remote sensing of SWE assessment with good temporal resolution (3 weeks) and a spatial scale (60–120 km) suited to regional studies.
